# Linezolid toxicity in patients with drug-resistant tuberculosis: a prospective cohort study

**DOI:** 10.1093/jac/dkac019

**Published:** 2022-02-02

**Authors:** Sean Wasserman, James CM Brust, Mahmoud T. Abdelwahab, Francesca Little, Paolo Denti, Lubbe Wiesner, Neel R. Gandhi, Graeme Meintjes, Gary Maartens

**Affiliations:** 1 Wellcome Centre for Infectious Diseases Research in Africa, Institute for Infectious Disease and Molecular Medicine, University of Cape Town, Cape Town, South Africa; 2 Division of Infectious Diseases and HIV Medicine, Groote Schuur Hospital, University of Cape Town, Cape Town, South Africa; 3 Division of General Internal Medicine, Department of Medicine, Albert Einstein College of Medicine & Montefiore Medical Center, Bronx, NY, USA; 4 Division of Clinical Pharmacology, University of Cape Town, Cape Town, South Africa; 5 Department of Statistical Sciences, University of Cape Town, Cape Town, South Africa; 6 Departments of Epidemiology & Global Health, Rollins School of Public Health, Emory University, Atlanta, GA, USA; 7 Division of Infectious Diseases, Department of Medicine, Emory School of Medicine, Emory University, Atlanta, GA, USA; 8 Department of Medicine, University of Cape Town, Cape Town, South Africa

## Abstract

**Background:**

Linezolid is recommended for treating drug-resistant tuberculosis. Adverse events are a concern to prescribers but have not been systematically studied at the standard dose, and the relationship between linezolid exposure and clinical toxicity is not completely elucidated.

**Patients and Methods:**

We conducted an observational cohort study to describe the incidence and determinants of linezolid toxicity, and to determine a drug exposure threshold for toxicity, among patients with rifampicin-resistant tuberculosis in South Africa. Linezolid exposures were estimated from a population pharmacokinetic model. Mixed-effects modelling was used to analyse toxicity outcomes.

**Results:**

151 participants, 63% HIV-positive, were enrolled and followed for a median of 86 weeks. Linezolid was permanently discontinued for toxicity in 32 (21%) participants. Grade 3 or 4 linezolid-associated adverse events occurred in 21 (14%) participants. Mean haemoglobin concentrations increased with time on treatment (0.03 g/dL per week; 95% CI, 0.02 to 0.03). Linezolid trough concentration, male sex, and age (but not HIV-positivity) were independently associated with a decrease in haemoglobin > 2 g/dL. Trough linezolid concentration of 2.5 mg/L or higher resulted in optimal model performance to describe changing haemoglobin and treatment-emergent anaemia (adjusted odds ratio 2.9; 95% CI, 1.3 to 6.8). Single nucleotide polymorphisms 2706A>G and 3010G>A in mitochondrial DNA were not associated with linezolid toxicity.

**Conclusions:**

Permanent discontinuation of linezolid was common, but linezolid-containing therapy was associated with average improvement in toxicity measures. HIV co-infection was not independently associated with linezolid toxicity. Linezolid trough concentration of 2.5 mg/L should be evaluated as a target for therapeutic drug monitoring.

## Introduction

Rifampicin-resistant tuberculosis (RR-TB) accounts for an expanding proportion of incident global TB cases and is an ongoing threat to EndTB targets. ^1^ Linezolid is a repurposed oxazolidinone antimicrobial with bactericidal activity against *M*. *tuberculosis*. ^2^ Inclusion of linezolid in treatment regimens for RR-TB is associated with treatment success and mortality reduction; ^3, 4^ as a result, WHO guidelines now recommend linezolid as a preferred agent for RR-TB. ^5^

The major drawback of linezolid is binding to human mitochondrial 16S ribosomal RNA (rRNA), which has a homologous structure to the *M*. *tuberculosis* target site, ^6^ resulting in dose-related mitochondrial toxicity that manifests most commonly as bone marrow suppression and peripheral neuropathy. These toxic effects may be treatment-limiting. ^7, 8^ The incidence and risk factors for linezolid toxicity have not been systematically studied in TB programs, ^9^ particularly in populations from sub-Saharan Africa with high rates of HIV co-infection, which could increase the risk of toxicity. ^10^ Factors possibly associated with linezolid toxicity include age, sex, and polymorphisms in mitochondrial DNA (mtDNA). ^11-13^ Overlapping complications from comorbidities (e.g peripheral neuropathy secondary to diabetes, HIV, or alcohol abuse) may also contribute. Estimating frequency and identifying risk profiles for serious linezolid toxicity will support deployment of this drug in programmatic settings.

An approach to mitigate toxicity is through optimized linezolid dosing, which requires characterization of the exposure-toxicity relationship. ^14^ Standard linezolid dosing in RR-TB (600 mg daily) is likely to achieve an *in vitro* efficacy target for *M tuberculosis* and reduce the emergence of resistance. ^15^ Trough concentrations are inversely correlated with mitochondrial function, ^16^ haemoglobin concentration in mouse models, ^17, 18^ and clinical toxicity among patients with Gram-positive infection. ^12, 19^ Pharmacokinetic (PK)-toxicity targets have been suggested from small clinical studies, but these are not adequately established for patients with TB. ^12, 16^ Linezolid trough concentrations correlate with area under the concentration-time curve (AUC, the target PK parameter for efficacy), ^20^ suggesting a potential role for therapeutic drug monitoring (TDM) if a concentration threshold target for clinical toxicity is defined. ^21^

We aimed to describe the incidence and determinants of linezolid toxicity, and to determine a drug exposure threshold for toxicity, among patients with RR-TB in a programmatic setting with a high HIV burden.

## Patients and Methods

### Design and population

This analysis was nested in a prospective observational cohort study (PROBeX) conducted at 3 drug-resistant TB referral hospitals in South Africa. The parent PROBeX study recruited 195 adults with known HIV status and culture-confirmed RR-TB who were initiating treatment with a bedaquiline-containing regimen between April 2016 and March 2018. ^22^ During the study period local treatment guidelines recommended an 18- to 24-month regimen. Linezolid was provided at a dose of 600 mg daily, with reduction to 300 mg daily at the discretion of treating clinicians if toxicity developed. Linezolid was recommended for the full treatment course if tolerated, but the duration was determined by treating clinicians. Treatment decisions were informed by clinical assessments and routine toxicity screening which included monthly full blood counts; linezolid TDM was not performed.

### Procedures

Participants were followed until 6 months after completion of therapy, or up to 24 months after study entry, at the start of bedaquiline therapy. Study visits occurred monthly during the first 6 months of therapy, then 6-monthly until study exit. Phlebotomy was performed at every visit for full blood count and lactate; results of these tests performed in routine care outside of study visits were also obtained. The modified Brief Peripheral Neuropathy Scale (BPNS) was used to screen for peripheral neuropathy. ^23^ We assessed visual acuity using logMAR charts and colour vision using 14-plate Ishihara charts to screen for optic neuropathy. Neuropathy screening was done at every study visit. Abnormal findings from laboratory and bedside testing were shared with treating clinicians in real time. Mitochondrial DNA (mtDNA) was extracted from stored whole blood and the 16S rRNA gene sequenced to detect two SNPs (2706A>G and 3010G>A) previously associated with linezolid-induced mitochondrial toxicity (methods in supplementary text). ^11^

### PK data

We did intensive PK sampling (pre- and at 1, 2, 3, 4, 5, 6, and 24 hours post-dose) on a subgroup of 21 participants at month 2 and sparse (pre-dose) PK sampling for the full cohort at months 1, 2, and 6 after initiation of linezolid therapy. Linezolid concentrations were measured in the Division of Clinical Pharmacology at the University of Cape Town using a validated liquid chromatography-tandem mass spectrometry (LC-MS/MS) assay. ^20^ We developed a population PK model using these data and derived average linezolid area under the concentration-time curve over 24 hours (AUC_0-24_) and trough values for individual participants, based on body weight and time-varying linezolid dose. ^24^

### Outcome definitions

The main outcome was linezolid toxicity measured by cytopenia, peripheral and optic neuropathy, and hyperlactatemia. We defined anaemia, thrombocytopenia, leukopenia, and hyperlactatemia according to Division of AIDS (DAIDS) Table for Grading the Severity of Adult and Pediatric Adverse Events; Version 2.1. Peripheral neuropathy was graded according to the modified BPNS score ^25^. Optic neuropathy was defined as an increase of 0.3 on the logMAR score in either eye ^26^ or a reduction in colour vision score of > 2. ^27^ We performed exploratory data analysis to identify thresholds for toxicity measures by observing distribution and trends of haematological parameters and lactate over time and relationship with baseline values. Early discontinuation of linezolid was defined as a permanent stop prior to 6 months of therapy.

### Analysis

Kaplan-Meier survival curves were computed to analyse and plot the timing of event onset; median times were reported for participants who experienced events. The primary outcome of interest was change in haemoglobin concentration from baseline. The key covariates were linezolid exposure, duration on linezolid, age, sex, and HIV status. Effect of risk-associated mtDNA SNPs was also explored. To describe changing continuous outcomes, we fitted linear mixed-effects regression models incorporating baseline controlling variables and time-varying covariates (linear time effect and linezolid exposure). We used conditional logistic regression for repeated toxicity events and computed marginal probabilities to represent risk; this approach was selected to incorporate multiple recuring events and account for within-individual correlation through inclusion of participant-specific random effects. ^28^ Internal model validation was performed using a k-fold cross-validation procedure. ^29^ We performed a piecewise (broken stick) regression procedure to identify the optimal threshold value of linezolid exposure that predicted clinical toxicity based on best model fit of linear regression models as measured by Akaike Information Criteria at multiple values of linezolid trough concentrations. ^30, 31^

The study was not formally powered as the predictors of linezolid toxicity or PK-pharmacodynamic relationships are not well characterized. A *post hoc* power calculation showed that a sample size of around 150 participants would have > 95% probability of detecting a 95% confidence interval with precision (width) of at least 0.18 for anaemia, given a standard deviation of 0.51 in our sample.

All analyses were performed with Stata/BE 17.0 (Statacorp).

### Ethics

This study was approved by the Human Research Ethics Committee at the University of Cape Town (437/2016), Albert Einstein College of Medicine, and Emory University. All participants provided written informed consent prior to performance of study procedures. The study was conducted and reported according to STROBE guidelines.

## Results

### Characteristics of study population

We included 151 participants out of 195 enrolled in the parent cohort; 44 were excluded because of no documented linezolid prescription (n = 38) or absent toxicity measure after starting linezolid (n = 6). Baseline characteristics are shown in (Table 1); 63% were HIV-positive, and 66% had fluoroquinolone-resistant TB. In addition to linezolid, all participants received bedaquiline; clofazimine, levofloxacin, pyrazinamide, terizidone, and para-aminosalicylic acid were provided to over 95%; and ethambutol was prescribed for 74 participants (49%). Prior to starting linezolid, the median haemoglobin was 11.8 g/dL (range 6.4 – 17.9). Median follow up from start of linezolid therapy was 86 weeks (range 3 - 183). A single A>G substitution at position 2706 was detected in 124 (87%) participants; no SNPs were detected at position 3010.

### Linezolid therapy and PK

**Figure 1:**
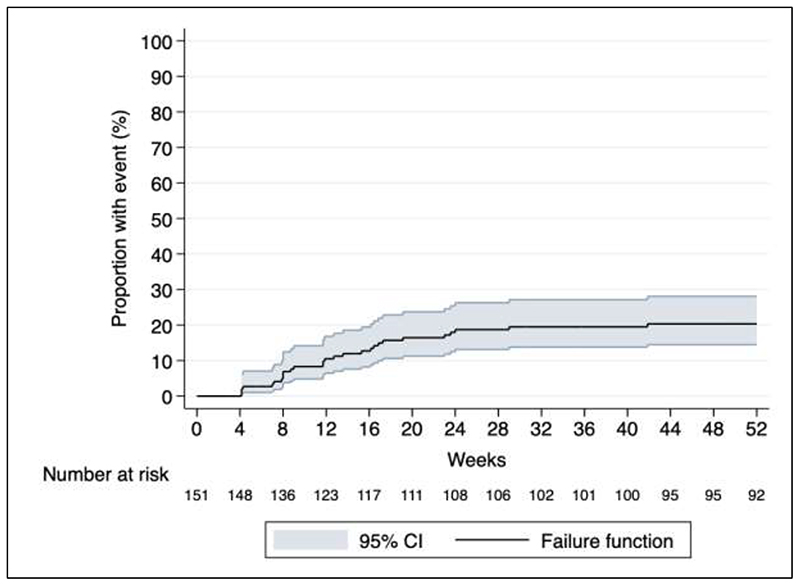
Time to early discontinuation of linezolid at 52 weeks. Kaplan-Meier plot with survival estimates for early discontinuation of linezolid, defined as permanent stop prior to completing 6 months of therapy.

The starting linezolid dose was 600 mg daily in 148 participants and 300 mg in three. The median duration of linezolid therapy, excluding treatment interruptions, was 336 days (IQR 159 – 506; range 6 - 862). Linezolid dose was reduced for 31 (21%) participants at a median time of 69 days (IQR 36 – 147). Linezolid was permanently discontinued in 32 (21%) participants at a median time of 60 days (IQR 20 - 99); 10 (31%) patients had either dose reduction or interruption prior to early discontinuation (Table 2 and Figure 1).

The individual PK parameters were derived from a population PK model based on observed concentrations for 95 participants and were predicted (based on weight and dose) for the other 56 participants with no measured linezolid concentrations. Median linezolid AUC_0-24_ was 168.9 mg∙h/L (IQR 143 – 194) and trough concentration was 2.1 mg/L (1.8 – 2.3) for the 600 mg dose (supplementary figure S1). There was an exponential relationship between AUC_0-24_ and trough concentrations, which were highly correlated (supplementary figure S2).

### Linezolid toxicity events

**Figure 2:**
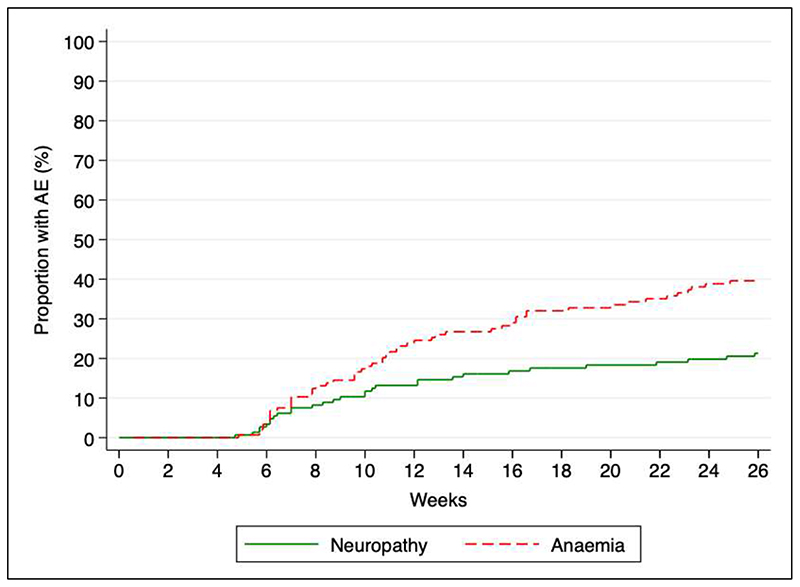
Time to development of any grade adverse event for anemia or peripheral neuropathy. Kaplan-Meier plot with superimposed individual survival estimates for anemia and neuropathy. Anemia defined as any new grade on DAIDS grading and neuropathy defined as any new grade on BPNS score. AE, adverse event.

Cumulative incidence of any new grade anaemia or peripheral neuropathy DAIDS event at 6 months was 39% (95% CI, 31 – 47) and 20% (95% CI, 14 – 27), respectively, with similar median time to experiencing the event: 11 weeks (IQR 7 - 17) for anaemia and 10 weeks (IQR 7 - 23) for neuropathy (Figure 2). New grade 3 or 4 events occurred in 21 participants: cumulative incidence 14% (95% CI, 9 - 21) at 6 months. 16 participants had reductions in visual acuity with a cumulative incidence of 12% (95% CI, 8 – 20) at 24 months; median time to onset was 10 weeks (range 5 - 79 weeks). Linezolid was dose-reduced or permanently discontinued in 5 participants with reduced visual acuity. Only one participant experienced reduction in colour vision (Table 3).

Additional toxicity outcomes were defined based on the observed data: anaemia, haemoglobin reduction > 2 g/dL; thrombocytopenia, platelet reduction > 250 x 10^9^/L; leukopenia, white cell count reduction > 4 cells x 10^9^/L; and hyperlactatemia, lactate increase > 1.5 mmol/L (Supplemental Table S1). Using these definitions, cumulative incidence of anaemia at 6 months was 33% (95% CI, 26 - 41), thrombocytopenia 16% (95% CI, 11 - 23), leukopenia 20% (95% CI, 14 - 28), and hyperlactatemia 15% (95% CI, 10 - 22).

### Relationship between linezolid exposure and toxicity

**Figure 3:**
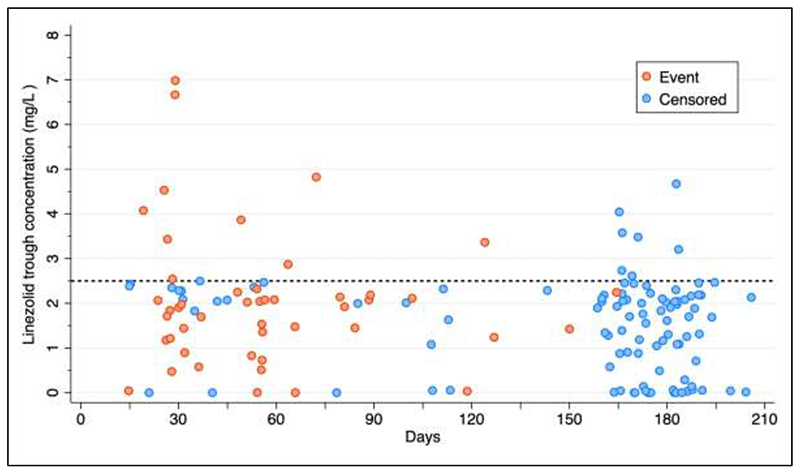
Observed relationship between anemia events and linezolid trough concentrations during the first 6 months of linezolid therapy. Events defined as reduction in hemoglobin > 2 g/dL in red circles; censoring at 6 months without anemia, and for lost to follow up, and death in blue circles. Dashed line indicates trough concentration of 2.5 mg/L.

A linezolid trough concentration of 2.5 mg/L resulted in optimal model fit to describe association with change in haemoglobin compared to other breakpoint values using piecewise regression (supplemental Table S2 and Figure S3). There was a clear time trend for the onset of anaemia, defined as a drop in haemoglobin > 2 g/dL, during the first 6 months of linezolid therapy: of the 47 participants who experienced anaemia, 43 (91%) events occurred within 120 days. 8 out of 9 (89%) participants with a linezolid trough concentration above 2.5 mg/L in this period had anaemia; 38% (21/55) with trough concentrations below this threshold had no anaemia (Figure 3).

### Factors associated with linezolid toxicity measures

**Figure 4:**
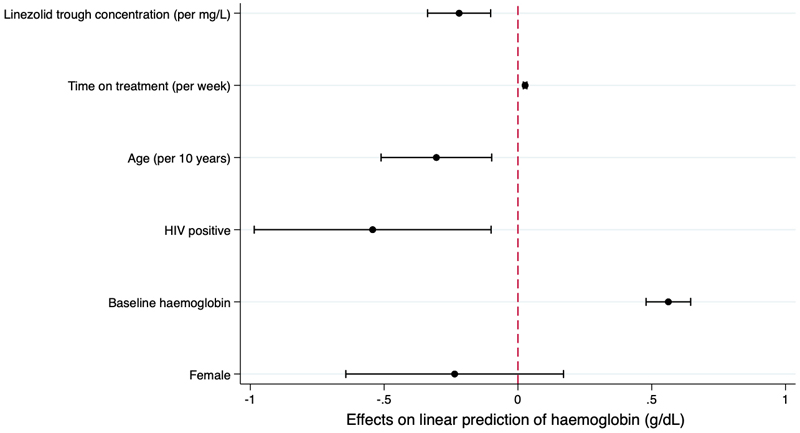
Predictors of longitudinal hemoglobin measures over the study period Estimates of mean effects on hemoglobin from the mixed-effects linear regression model. Dots indicate point estimate; black lines indicate 95% confidence interval; dashed red line indicates no effect.

Mean haemoglobin was predicted to increase with time on treatment (0.03 g/dL per week; 95% CI, 0.02 to 0.03) and with a higher pre-treatment haemoglobin (0.6 g/dL; 95% CI, 0.5 to 0.7); and to decrease with increasing linezolid trough concentrations (-0.2 g/dL per 1 mg/L; 95% CI, -0.3 to -0.1), HIV-positivity (-0.5 g/dL; 95% CI, -1.0 to -0.1), and age (-0.3 g/dL per 10 years; 95% CI, -0.5 to -0.1) (Figure 4; supplemental S4A). 31% of total variability was due to inter-individual variability. Model-predicted haemoglobin at 4 weeks was 8.2 g/dL (95% CI, 7.8 to 8.8) for an HIV-positive participant with the lowest pre-treatment haemoglobin of 6.4 g/dL (at observed values of other parameters).

Average platelet count, white blood cell count, and lactate decreased over time when adjusted for baseline values, HIV status, age, gender, and linezolid trough concentrations (supplement S4B-D). Lactate increase was associated with linezolid trough concentrations (0.08 mmol/L increase per 1 mg/L linezolid trough, 95% CI 0.01 to 0.2). There was an inverse association between linezolid trough concentrations and both platelet count (-11.4 × 10^9^/L, 95% CI -19.7 to -3.1) and white cell count (-0.2 cells × 10^9^/L, 95% CI -0.3 to -0.02). Sensitivity analysis was done for all outcomes including only PK estimates from measured concentrations, without substantial change in parameter estimates.

**Figure 5:**
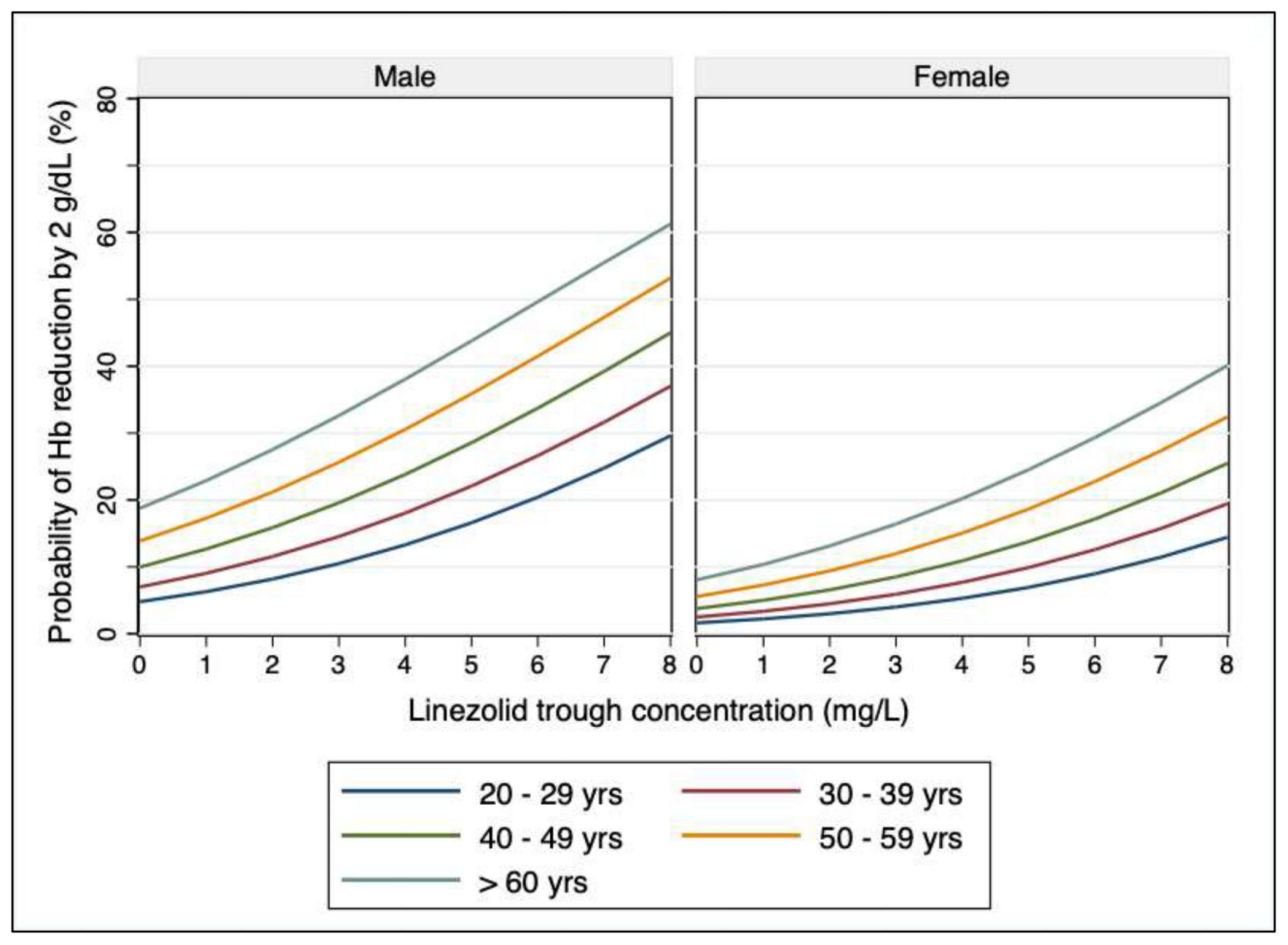
Predicted probability of anemia by sex. Marginal predictions from mixed-effects logistic regression model for probability of anemia, defined as reduction in hemoglobin (Hb) ≥ 2 g/dL. Colored lines indicate age ranges, defined in the legend.

Factors independently associated with anaemia, defined as a reduction in haemoglobin > 2 g/dL, were linezolid trough concentration (aOR 1.4 per 1 mg/L increase, 95% CI 1.1 to 1.8), male sex (aOR 3.4, 95% CI 1.5 to 8.1) and age (aOR 1.7 per 10-year increase, 95% CI 1.2 to 2.3). HIV-positivity was not a significant predictor (aOR 1.2, 95% CI 0.5 to 2.9). There was large inter-individual variability (rho = 0.47). Marginal predictions for probability of anaemia are shown in Figure 5. A linezolid trough concentration ≥ 2.5 mg/L was associated with 2.9-fold increased odds (95% CI, 1.3 to 6.8) of anaemia in the adjusted model. There was also a significant association between linezolid trough concentration and thrombocytopenia and hyperlactatemia (supplemental tables S3 and S4), but not with neuropathy (supplemental table S5). There was no effect modification with inclusion of the mtDNA A2706G mutation in any model (data not shown). Model performance and parameter estimates were similar for all toxicity outcomes when AUC_0-24_ was tested instead of trough concentration (data not shown).

## Discussion

In this cohort of South African RR-TB patients with an HIV prevalence of 63%, mild anaemia and peripheral neuropathy occurred frequently, and linezolid was prematurely discontinued in a fifth of patients. However, severe adverse events were infrequent, and on average, linezolid use in a multidrug regimen was associated with a positive treatment effect on haemoglobin over time. We identified a trough concentration threshold that predicted higher risk of anaemia, the most specific measure of linezolid toxicity, which, if validated, could be used for TDM.

Linezolid-associated haematological and neurological toxicity is a major concern for prescribers. ^32^ The most recent systematic review, published in 2015, summarized data from 14 retrospective studies and 1 randomized controlled trial; all but one study included fewer than 50 patients, and there was large heterogeneity in outcome definitions and treatment. The pooled proportion of adverse events leading to linezolid discontinuation was 29%, with anaemia and peripheral neuropathy reported in 31% and 27%, respectively. ^7^ Importantly, none of the included studies were conducted in Africa where high rates of HIV co-infection and limited monitoring capability may exacerbate the risk of linezolid toxicity. ^10^ A recent small prospective study (n = 63) among South African patients with RR-TB and a high HIV prevalence described similar proportions with anaemia and neuropathy at the 600 mg dose, with linezolid interruption or discontinuation in 35%, but severity was not reported, and it is unclear how outcomes were ascertained. ^9^

To obtain more reliable estimates of toxicity we defined haematological events using the established DAIDS grading system and used the validated BPNS scale to screen for peripheral neuropathy. We identified incident severe (Grade 3 or 4) adverse events in 15% of our participants. Anaemia followed by mild peripheral neuropathy were the most common adverse events, which is in line with other TB studies. ^3, 8^ Most adverse events occurred within the first 4 months of therapy, with similar timing of onset for anaemia and neuropathy at a median of around 10 weeks. Neuropathy has occurred relatively later than myelosuppression in some studies, leading to suggestions of a duration-dependent effect for neurotoxicity. ^3, 33, 34^ However, these studies were limited by small size and lack of consistent outcome definitions, and there is no clear biological explanation for this hypothesis. The onset of peripheral neuropathy in the Nix-TB trial, which used a higher dose of linezolid, occurred mainly in the initial 3 months of treatment, consistent with our findings. ^8^ Cumulative incidence of reduced visual acuity was 12% at 24 months in our cohort, the earliest detected at 5 weeks after starting linezolid. This is within the range reported from other studies ^7^ but is likely an overestimate of true linezolid-induced optic neuropathy because visual acuity testing lacks specificity, ^35^ and many participants were on concomitant ethambutol which can also cause ocular toxicity. In the Nix-TB trial optic nerve disorders were suspected in 11.9% by bedside testing but confirmed optic neuropathy only occurred in 2 (<2%) participants. ^8, 36^

There were no Grade 3 or 4 thrombocytopenia events in our cohort. Platelets are acute phase reactants, increasing in response to systemic inflammation, including from TB, ^37, 38^ while haemoglobin changes in the opposite direction. ^39^ The negative correlation between platelet counts and haemoglobin over time in our data suggests that reductions in platelets represent reduction in systemic inflammation due to treatment rather than linezolid toxicity. Therefore, platelets are not a good pharmacodynamic marker for linezolid toxicity in tuberculosis.

Average haemoglobin increased over time after adjustment for other factors. HIV positivity was independently associated with reduced haemoglobin but not with anaemia (reductions > 2 g/dL). This effect was likely related to underlying HIV-related myelosuppression, as only 2 participants were on zidovudine. In the Nix-TB trial there was also no increase in linezolid-associated adverse events among HIV-positive participants. ^8^ Independent predictors of anaemia in our cohort were age, male sex, and linezolid trough concentrations, which have been associated with linezolid toxicity in patients with Gram-positive infections. ^12, 13^ In our cohort, the predicted probability of substantial haemoglobin reduction was ~10% for male participants at the median values of age and linezolid trough concentrations (Fig. 5), indicating relative safety of the 600 mg daily dose in our population. Despite this, linezolid was interrupted, dose reduced, or discontinued early in over half of participants, suggesting either the presence of unmeasured adverse events or a low threshold by clinicians to alter or stop therapy due to concerns about toxicity potential. ^32^

Hyperlactatemia is a complication of linezolid therapy due to mitochondrial injury, ^40^ and there have been case reports of lactic acidosis. ^41, 42^ Data is scarce on the incidence of hyperlactatemia in cohort studies. In the Nix-TB trial there were only 8 cases (3 had lactic acidosis), much lower than the 30% incidence in our study. ^8, 36^ Possible reasons for this discrepancy include technical issues relating to sample processing, a sicker population in our study, and different definitions of hyperlactatemia. Nonetheless, there were relatively few severe events, with only 12 Grade 2 episodes and 15% with increases > 1.5 mmol/L at 6 months; on average, lactate decreased over time on linezolid therapy.

The presence of SNPs at positions 2706 and 3010 in mitochondrial 16S rRNA have been reported in association with hyperlactatemia during linezolid therapy and are hypothesized to confer genetic susceptibility to linezolid toxicity through enhanced binding to mitochondrial structures. ^11^ The G3010A SNP was not detected in any of our participants and the presence of A2706G was not associated with any toxicity measure, corroborating findings from a trial among Korean DR-TB patients. ^16^

Linezolid is a good candidate for TDM in RR-TB because of its narrow therapeutic margin and large inter-individual variability. ^20, 24^ Linezolid trough concentrations are consistently associated with haematological toxicity measures, ^12, 16-18, 43^ including in our cohort. A trough threshold of 2 mg/L has been suggested based on the high proportion of clinical events observed above that value among Korean XDR-TB patients in a small trial (n = 38). ^16^ Although this target is now widely applied in PK/PD analyses, the specificity is poor, and it has not been validated in other cohorts. Using a model-based approach we found that a trough concentration of ≥ 2.5 mg/L described change in haemoglobin better than other tested values and had a large effect on risk of significant haemoglobin drop after adjustment for other factors - this finding has potential for use in TDM to reduce risk of adverse events. Where TDM is unavailable close clinical and haematologic (especially haemoglobin) monitoring could trigger linezolid dose changes once toxicity develops. ^44^

There are limitations to consider when interpreting our findings. There was no planned phlebotomy or neuropathy screening in the first month of our study, which may have contributed to the low event rate observed within the first few weeks of linezolid. Although we obtained all full blood count results from routine care, bedside haemoglobin testing was not captured, neither were blood transfusions, potentially masking more severe anaemia. However, a strength of our study is that it reflects real world practice and outcomes. The observational nature of the study resulted in unbalanced visits and missing observations, but random effects models are valid under flexible missing data assumptions, including missingness at random, ^45^ supporting our conclusions. Linezolid concentrations were missing for a third of participants and individual drug exposures were predicted from a population PK model based on measured body weight and dose. The model was developed using rich data from a subgroup of participants in our cohort and, on sensitivity analysis, inclusion of sparse concentrations did not alter model performance. A limitation of using trough values is that they are strongly influenced by other model parameters, plus uncertainty in dosing timing.

We addressed this by including separate additive error and additive lag variability relative to reported time of the dose to account for uncertainty in unobserved dosing (affecting sparse samples). ^24^ Additionally, there was no effect modification on parameter estimates when only values with observed concentrations were included in toxicity outcome models. Finally, our study was not formally powered, influencing the precision of our estimates and ability to detect relationships with smaller effects. There were relatively few observations above our identified toxicity concentration threshold of 2.5 mg/L, emphasizing the need to validate this finding. However, our sample size is larger than that used in previous studies which successfully identified PK/PD relationships for first-line TB treatment ^46, 47^ and for other studies evaluating linezolid toxicity. ^9, 16^

In summary, we characterized linezolid toxicity in a DR-TB treatment program among patients with high HIV prevalence. Severe events were uncommon at the standard dose of 600 mg daily in this setting and overall, linezolid use was associated with improvement haemoglobin and other toxicity measures. A trough concentration threshold of 2.5 mg/L should be further evaluated as a potential target for TDM of this important TB drug.

## Supplementary Material

Supplementary material

## Figures and Tables

**Table 1:** Baseline characteristics

	n (%) or median (IQR)	Denominator
**Age, yrs**	34 (28-42)	151
**Female sex**	84 (56%)	151
**Ethnicity**		151
**Black**	127 (84%)	
**Mixed race**	22 (15%)	
**Other**	2 (1%)	
**Weight, kg**	53 (47-60)	150
**BMI, kg·m^-2^**	20(18-22)	149
**HIV-positive**	95 (63%)	151
**CD4 count, cells/mm^3^**	212 (111-438)	91
**Antiretroviral therapy**	81 (85%)	95
**Previously treated TB**	111 (74%)	151
**Resistance pattern of baseline isolate**		141
**MDR**	12 (9%)	
**Pre-XDR (injectable)**	36 (26%)	
**Pre-XDR (fluoroquinolone)**	32 (23%)	
**XDR**	61 (43%)	
**Creatinine, μmol/L**	62 (51-71)	150
**Creatinine clearance, mL/min**	105 (88-125)	149
**Haemoglobin, g/dL**	11.8 (10.4 – 13.2) Range: 6.4 – 17.9	148
**White blood cell count (x 10^9^ cells/L)**	7.3 (5.3 – 9.5) Range: 1.4 – 27.1	148
**Platelets (x 10^9^/L)**	345 (271 - 489) Range: 132 - 1131	148
**Venous lactate, mmol/L**	1.7 (1.3 – 2.3) Range: 0.8 – 6.5	115
**BPNS grade**		121
**0**	100 (83%)	
**1**	17 (14%)	
**2**	4 (3%)	
**LogMAR score (right)**	0 (range 0 - 1.0)	113
**LogMAR score (left)**	0 (range 0 - 0.8)	111
**Ishihara score**	6 (6-6)	123
**mtDNA 16S rRNA polymorphism**		
**2706A>G**	124 (87%)	142
**3010G>A**	0 (0)	142

BMI, body mass index; MDR, multi-drug resistant (resistance to rifampicin plus isoniazid);

XDR, extensively drug resistant (additional resistance to fluoroquinolones and injectable agents); pre-XDR (additional resistance to either fluoroquinolones or injectable agents); BPNS, modified brief peripheral neuropathy score.

**Table 2:** Details of linezolid interruption

Parameter	n = 151
Linezolid changes - Dose reduction - Interruption then dose reduction - Interruption then same dose - Early discontinuation	31 (21%) 6 (4%) 10 (7%) 32 (21%)
Linezolid duration - Until first interruption/change - Duration of interruption - Until early discontinuation - Total duration	69 days (IQR 36 – 147; range 4 – 530) 42 days (IQR 28 – 85; range 5 – 315) 60 days (IQR 20 – 99; range 12 – 179) 336 days (IQR 159 – 506; range 6 - 862)

Data are number (percent) or median (interquartile range). Early discontinuation defined as permanent discontinuation before 6 months. 10 (31%) patients had either dose reduction or interruption prior to early discontinuation. Total duration excludes time off linezolid during treatment interruptions.

**Table 3:** New adverse events after starting linezolid

	Number of participants with any event (n = 151)	Grade 1	Grade 2	Grade 3	Grade 4	Cumulative incidence of any grade at 6 months (95% CI)	Event rate (per 100 person-weeks) (95% CI)
**Anaemia**	58	24	14	13	6	39% (31 – 47)	0.8 (0.6 – 1.0)
**Thrombocytopenia**	10	8	2	0	0	6% (3 – 11)	0.1 (0.0 – 0.2)
**Leukopenia**	6	5	0	1	0	4% (2 – 9)	0.1 (0.0 – 0.2)
**Peripheral neuropathy**	37^a^	32	4	0	1	20% (14 – 27)	0.4 (0.3 – 0.5)
**Reduced visual acuity**	16^b^	-	-	-	-	9% (5 – 15)	0.1 (0.1 – 0.2)
**Worsening colour vision**	1^c^	-	-	-	-	-	-
**Hyperlactatemia**	51^d^	43	8	-	-	31% (24 – 40)	0.6 (0.5 – 0.8)

a n = 121

b n = 119

c n = 123

d n = 115

These data are for the highest-grade adverse event experienced by individual participants. Anaemia, thrombocytopenia, leukopenia, and hyperlactatemia were defined according to Division of AIDS (DAIDS) Table for Grading the Severity of Adult and Pediatric Adverse Events; Version 2.1. Data on grade 3 or 4 hyperlactatemia was not collected as associated symptoms were not ascertained and pH was not measured. Peripheral neuropathy was graded according to the modified BPNS score. Optic neuropathy was defined as an increase of 0.3 on the logMAR score in either eye or a reduction in color vision score of > 2 on a 14-plate Ishihara chart.

## References

[1] World Health Organization (2020). Global tuberculosis report 2020.

[2] Diacon AH, De Jager VR, Dawson R (2020). Fourteen-Day Bactericidal Activity, Safety, and Pharmacokinetics of Linezolid in Adults with Drug-Sensitive Pulmonary Tuberculosis. Antimicrob Agents Chemother.

[3] Lee M, Lee J, Carroll MW (2012). Linezolid for treatment of chronic extensively drug-resistant tuberculosis. N Engl J Med.

[4] Ahmad N, Ahuja SD, Akkerman OW (2018). Treatment correlates of successful outcomes in pulmonary multidrug-resistant tuberculosis: an individual patient data meta-analysis. The Lancet.

[5] World Health Organization (2019). WHO consolidated guidelines on drug-resistant tuberculosis treatment.

[6] De Vriese AS, Coster RV, Smet J (2006). Linezolid-induced inhibition of mitochondrial protein synthesis. Clin Infect Dis.

[7] Zhang X, Falagas ME, Vardakas KZ (2015). Systematic review and meta-analysis of the efficacy and safety of therapy with linezolid containing regimens in the treatment of multidrug-resistant and extensively drug-resistant tuberculosis. J Thorac Dis.

[8] Conradie F, Diacon AH, Ngubane N (2020). Treatment of Highly Drug-Resistant Pulmonary Tuberculosis. N Engl J Med.

[9] Olayanju O, Esmail A, Limberis J (2019). Linezolid interruption in patients with fluoroquinolone-resistant tuberculosis receiving a bedaquiline-based treatment regimen. Int J Infect Dis.

[10] Hughes J, Isaakidis P, Andries A (2015). Linezolid for multidrug-resistant tuberculosis in HIV-infected and -uninfected patients. Eur Respir J.

[11] Palenzuela L, Hahn NM, Nelson RP (2005). Does linezolid cause lactic acidosis by inhibiting mitochondrial protein synthesis?. Clin Infect Dis.

[12] Pea F, Viale P, Cojutti P (2012). Therapeutic drug monitoring may improve safety outcomes of long-term treatment with linezolid in adult patients. J Antimicrob Chemother.

[13] Takahashi Y, Takesue Y, Nakajima K (2011). Risk factors associated with the development of thrombocytopenia in patients who received linezolid therapy. J Infect Chemother.

[14] Wasserman S, Meintjes G, Maartens G (2016). Linezolid in the treatment of drug-resistant tuberculosis: the challenge of its narrow therapeutic index. Expert Rev Anti Infect Ther.

[15] Bolhuis MS, Akkerman OW, Sturkenboom MGG (2018). Linezolid-based Regimens for Multidrug-resistant Tuberculosis (TB): A Systematic Review to Establish or Revise the Current Recommended Dose for TB Treatment. Clin Infect Dis.

[16] Song T, Lee M, Jeon H-S (2015). Linezolid Trough Concentrations Correlate with Mitochondrial Toxicity-Related Adverse Events in the Treatment of Chronic Extensively Drug-Resistant Tuberculosis. EBioMedicine.

[17] Bigelow KM, Deitchman AN, Li SY (2021). Pharmacodynamic Correlates of Linezolid Activity and Toxicity in Murine Models of Tuberculosis. J Infect Dis.

[18] Bigelow KM, Tasneen R, Chang YS (2020). Preserved Efficacy and Reduced Toxicity with Intermittent Linezolid Dosing in Combination with Bedaquiline and Pretomanid in a Murine Tuberculosis Model. Antimicrob Agents Chemother.

[19] Cazavet J, Bounes FV, Ruiz S (2020). Risk factor analysis for linezolid-associated thrombocytopenia in critically ill patients. Eur J Clin Microbiol Infect Dis.

[20] Wasserman S, Denti P, Brust JCM (2019). Linezolid Pharmacokinetics in South African Patients with Drug-Resistant Tuberculosis and a High Prevalence of HIV Coinfection. Antimicrob Agents Chemother.

[21] Kamp J, Bolhuis MS, Tiberi S (2017). Simple strategy to assess linezolid exposure in patients with multi-drug-resistant and extensively-drug-resistant tuberculosis. Int J Antimicrob Agents.

[22] Brust JCM, Gandhi NR, Wasserman S (2021). Effectiveness and cardiac safety of bedaquiline-based therapy for drug-resistant tuberculosis: a prospective cohort study. Clin Infect Dis.

[23] McArthur JH (1998). The reliability and validity of the subjective peripheral neuropathy screen. J Assoc Nurses AIDS Care.

[24] Abdelwahab MT, Wasserman S, Brust JCM (2021). Linezolid population pharmacokinetics in South African adults with drug-resistant tuberculosis. Antimicrob Agents Chemother.

[25] Cherry CL, Wesselingh SL, Lal L (2005). Evaluation of a clinical screening tool for HIV-associated sensory neuropathies. Neurology.

[26] World Health Organization (2003). Consultation on development of standards for characterization of vision loss and visual functioning.

[27] Lee JK, Lee JY, Kim DK (2019). Substitution of ethambutol with linezolid during the intensive phase of treatment of pulmonary tuberculosis: a prospective, multicentre, randomised, open-label, phase 2 trial. Lancet Infect Dis.

[28] Hu FB, Goldberg J, Hedeker D (1998). Comparison of population-averaged and subject-specific approaches for analyzing repeated binary outcomes. Am J Epidemiol.

[29] Daniels B (2012). CROSSFOLD: Stata module to perform k-fold cross-validation. Statistical Software Components.

[30] McZgee VE, Carleton WT (1970). Piecewise Regression. J Am Stat Assoc.

[31] Muggeo VM (2003). Estimating regression models with unknown break-points. Stat Med.

[32] McDowell A, Haas M, Seaworth B (2021). Linezolid use for the treatment of multidrug-resistant tuberculosis, TB centers of excellence, United States, 2013-2018. J Clin Tuberc Other Mycobact Dis.

[33] Tang S, Yao L, Hao X (2015). Efficacy, safety and tolerability of linezolid for the treatment of XDR-TB: a study in China. Eur Respir J.

[34] Bolhuis MS, Tiberi S, Sotgiu G (2015). Linezolid tolerability in multidrug-resistant tuberculosis: a retrospective study. Eur Respir J.

[35] Dempsey SP, Sickman A, Slagle WS (2018). Case Report: Linezolid Optic Neuropathy and Proposed Evidenced-based Screening Recommendation. Optom Vis Sci.

[36] TB Alliance (2019). Pretomanid FDA Sponsor Briefing Document.

[37] Robson SC, White NW, Aronson I (1996). Acute-phase response and the hypercoagulable state in pulmonary tuberculosis. Br J Haematol.

[38] Baynes RD, Bothwell TH, Flax H (1987). Reactive thrombocytosis in pulmonary tuberculosis. J Clin Pathol.

[39] Minchella PA, Donkor S, Owolabi O (2015). Complex Anemia in Tuberculosis: The Need to Consider Causes and Timing When Designing Interventions. Clin Infect Dis.

[40] Garrabou G, Soriano A, Lopez S (2007). Reversible inhibition of mitochondrial protein synthesis during linezolid-related hyperlactatemia. Antimicrob Agents Chemother.

[41] Boutoille D, Grossi O, Depatureaux A (2009). Fatal lactic acidosis after prolonged linezolid exposure for treatment of multidrug-resistant tuberculosis. Eur J Intern Med.

[42] Scotton P, Fuser R, Torresan S (2008). Early linezolid-associated lactic acidosis in a patient treated for tuberculous spondylodiscitis. Infection.

[43] Pea F, Furlanut M, Cojutti P (2010). Therapeutic drug monitoring of linezolid: a retrospective monocentric analysis. Antimicrob Agents Chemother.

[44] Imperial MZ, Nedelman JR, Conradie F (2021). Proposed linezolid dosing strategies to minimize adverse events for treatment of extensively drug-resistant tuberculosis. Clin Infect Dis.

[45] Little RJ, Rubin DB (2019). Statistical analysis with missing data.

[46] Pasipanodya JG, McIlleron H, Burger A (2013). Serum drug concentrations predictive of pulmonary tuberculosis outcomes. J Infect Dis.

[47] Chigutsa E, Pasipanodya JG, Visser ME (2015). Impact of nonlinear interactions of pharmacokinetics and MICs on sputum bacillary kill rates as a marker of sterilizing effect in tuberculosis. Antimicrob Agents Chemother.

