# Supplementary material for: Linezolid toxicity in patients with drug-resistant tuberculosis: a prospective cohort study
Source: J Antimicrob Chemother. Author manuscript; Available in PMC 2022 Mar 31. (PMC7612559; doi:10.1093/jac/dkac019)
Supplement: Supplementary material [file EMS140905-supplement-Supplementary_material.docx]

**Linezolid toxicity in patients with drug-resistant tuberculosis: a prospective cohort study**

Sean Wasserman, James CM Brust, Mahmoud T Abdelwahab, Francesca Little, Paolo Denti, Lubbe Wiesner, Neel R Gandhi, Graeme Meintjes, Gary Maartens

**ONLINE DATA SUPPLEMENT**

**Contents**

| **Label** | **Title** | **Page** |
| --- | --- | --- |
| Figure S1 | Linezolid secondary pharmacokinetic parameters, by dose. | 2 |
| Figure S2 | Correlation between linezolid AUC and trough concentrations. | 2 |
| Figure S3 | Change in hemoglobin from baseline values by linezolid trough concentration. | 3 |
| Table S1 | Toxicity outcomes defined from observed data | 3 |
| Table S2 | Akaike Information Criteria estimates for range of linezolid trough spline terms in mixed-effects linear regression model for change in hemoglobin (Hb). | 4 |
| Figure S4  S4 (A)  S4 (B)  S4 (C)  S4 (D) | Change in toxicity measures over time.  Hemoglobin  Platelet count  White cell count  Lactate | 5  5  6  6 |
| Table S3 | Conditional logistic regression model outputs for thrombocytopenia, defined as reduction in platelet count by ≥ 250 x10^9^/L. | 7 |
| Table S4 | Conditional logistic regression model outputs for hyperlactatemia, defined as increase in lactate concentration by ≥ 1.5 mmol/L. | 7 |
| Table S5 | Conditional logistic regression model outputs for peripheral neuropathy, defined as any new Grade increase on Brief Peripheral Neuropathy Score. | 7 |
| Text | Methods for mitochondrial DNA extraction and sequencing | 8 - 9 |

**Figure S1. Linezolid secondary pharmacokinetic parameters, by dose.**

Distribution of linezolid area under the concentration-time curve (AUC), left panel, and trough concentrations, right panel, by dose. Estimates derived from population pharmacokinetic model. Open circles are individual values for each study visit, boxes indicate median and interquartile ranges, whiskers indicate upper adjacent value (1.5x IQR). Dashed red line indicates trough value of 2 mg/L, the putative toxicity threshold derived elsewhere.

**Figure S2. Correlation between linezolid AUC and trough concentrations.**

Secondary pharmacokinetic parameters derived from population pharmacokinetic model. AUC, area under the concentration-time curve; 95% CI, confidence interval.

**
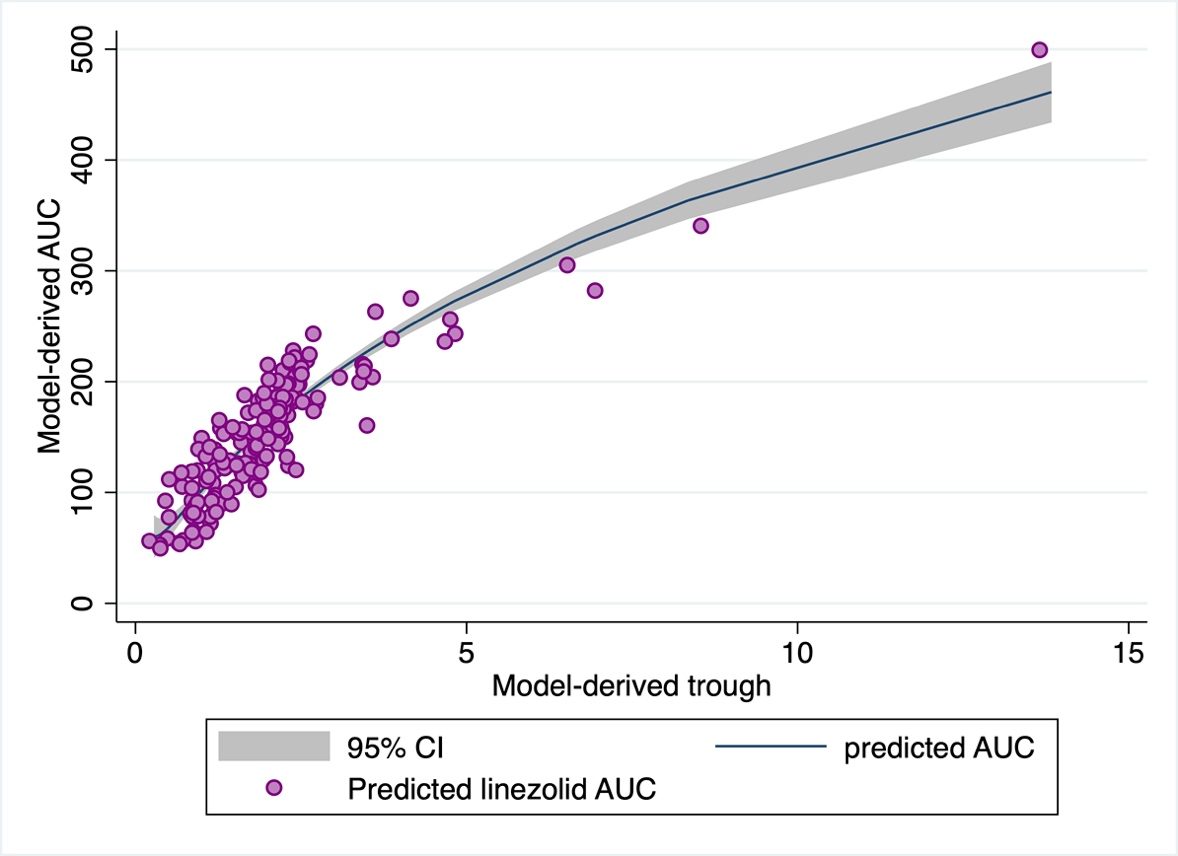
**

**Figure S3. Change in hemoglobin from baseline values by linezolid trough concentration.**

Mixed-effects linear regression model fit for change in hemoglobin with spline term for trough concentration at 2.5 mg/L, demonstrating model fit to the data. Blue dots indicate observed values, solid line indicates locally weighted smoothing, dashed lines indicate model-predictions.


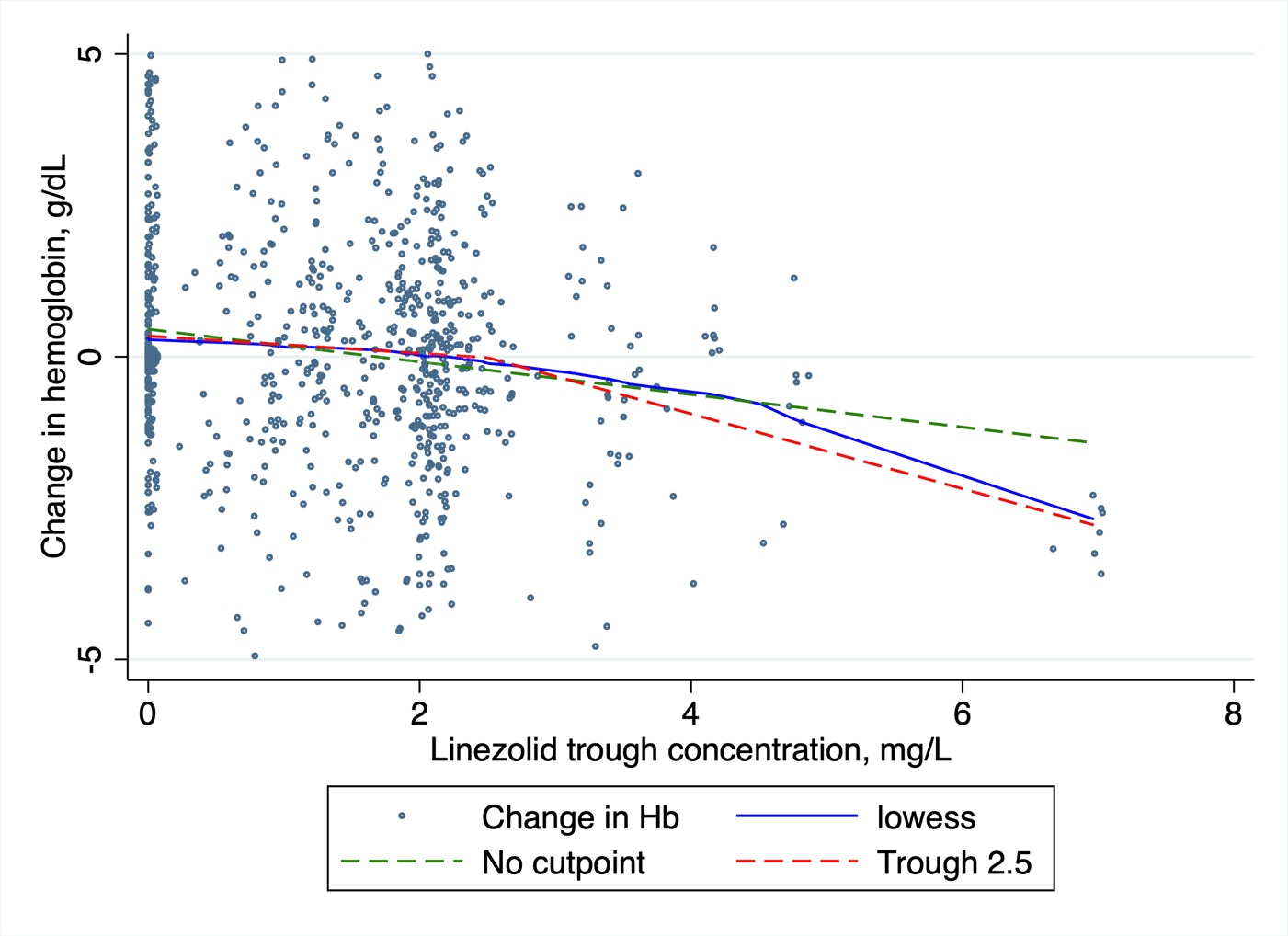


**Table S1. Toxicity outcomes defined from observed data**

| **Toxicity outcome** | **Measure** | **Absolute change** | **Percentile** | **Percentage change** | **Percentile** |
| --- | --- | --- | --- | --- | --- |
| Anaemia | Haemoglobin, g/dL | - 2 | 25 | -20 | 10 |
| Thrombocytopenia | Platelet count, x10^9^/L | -250 | 10 | -50 | 10 |
| Leukopenia | White cell count, x10^9^/L | -4 | 10 | -50 | 10 |
| Hyperlactataemia | Lactate, mmol/L | +1.5 | 5 | +25 | 10 |

**Table S2. Akaike Information Criteria estimates for range of linezolid trough spline terms in mixed-effects linear regression model for change in haemoglobin (Hb).**

Trough value 2.5 mg/L had the lowest AIC and discriminated best for observed events (Figure 3 in main text).

| **Model (trough cutoff)** | **AIC** |
| --- | --- |
| No spline term | 3805.219 |
| 1.0 mg/L | 3801.814 |
| 1.1 mg/L | 3801.091 |
| 1.2 mg/L | 3800.48 |
| 1.3 mg/L | 3800.344 |
| 1.4 mg/L | 3800.467 |
| 1.5 mg/L | 3800.643 |
| 1.6 mg/L | 3800.698 |
| 1.7 mg/L | 3800.362 |
| 1.8 mg/L | 3800.5 |
| 1.9 mg/L | 3800.615 |
| 2.0 mg/L | 3800.682 |
| 2.1 mg/L | 3800.333 |
| 2.2 mg/L | 3800.411 |
| 2.3 mg/L | 3800.11 |
| 2.4 mg/L | 3799.834 |
| 2.5 mg/L | 3799.797 |
| 2.6 mg/L | 3800.131 |
| 2.7 mg/L | 3800.434 |
| 2.8 mg/L | 3800.706 |
| 2.9 mg/L | 3800.949 |
| 3.0 mg/L | 3801.048 |

**Figure S4. Change in toxicity measures over time.**

Teal lines indicate observed values, dark blue line indicates model-predicted trend (adjusted for covariates), blue shading indicates 95% confidence interval.

**S4 (A). Hemoglobin**

**
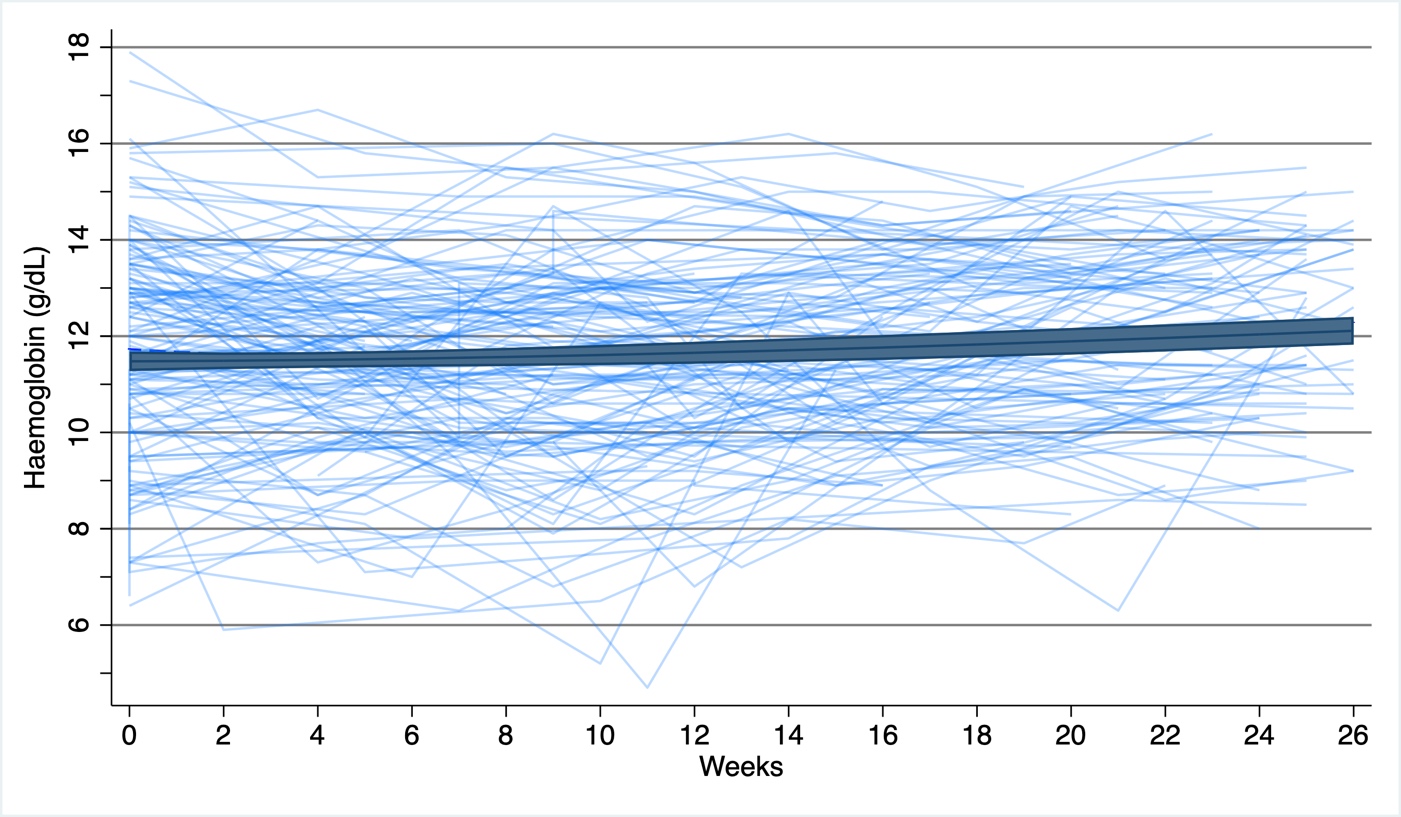
**

**S4 (B). Platelet count**

**
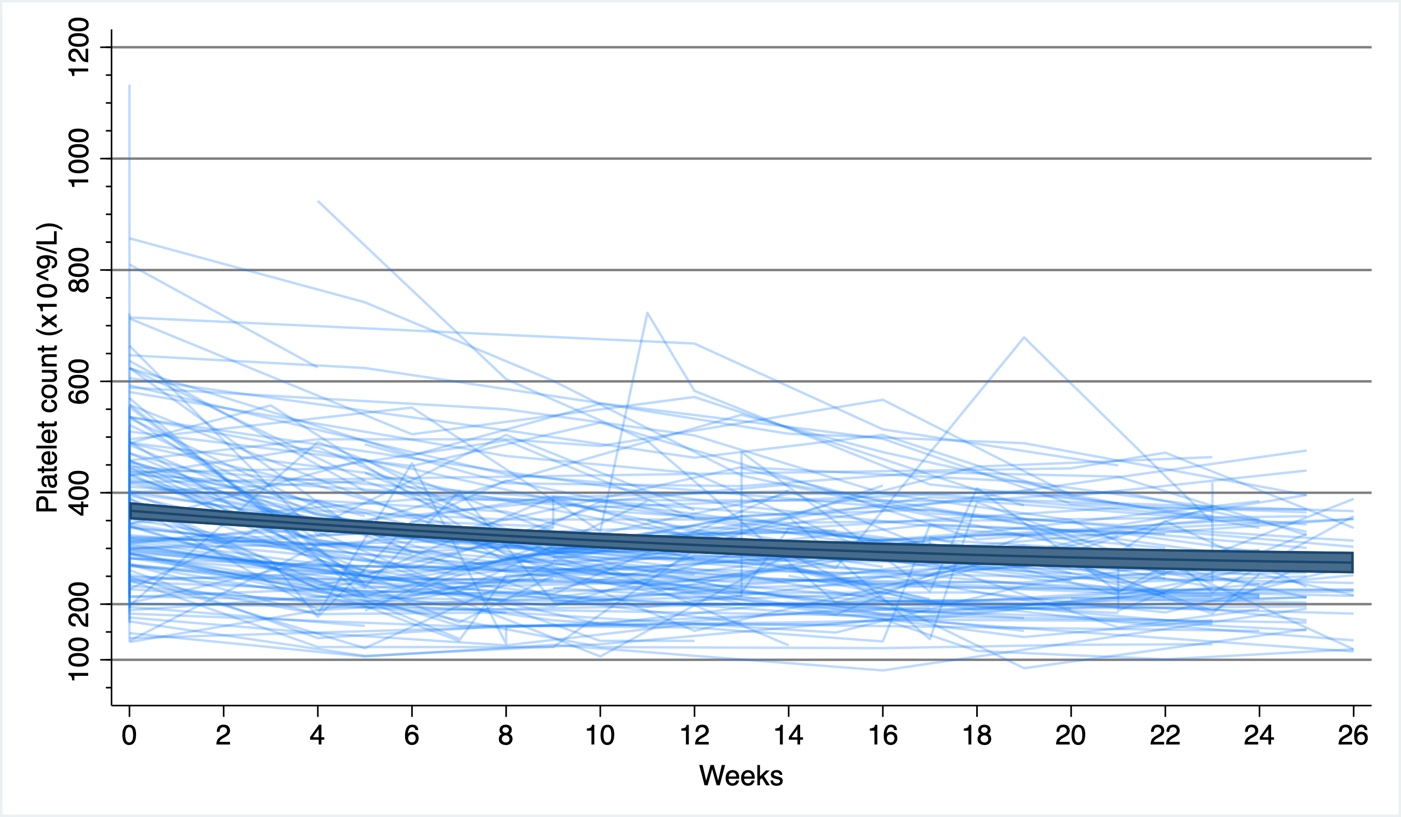
**

**S4 (C). White blood cell count**

**
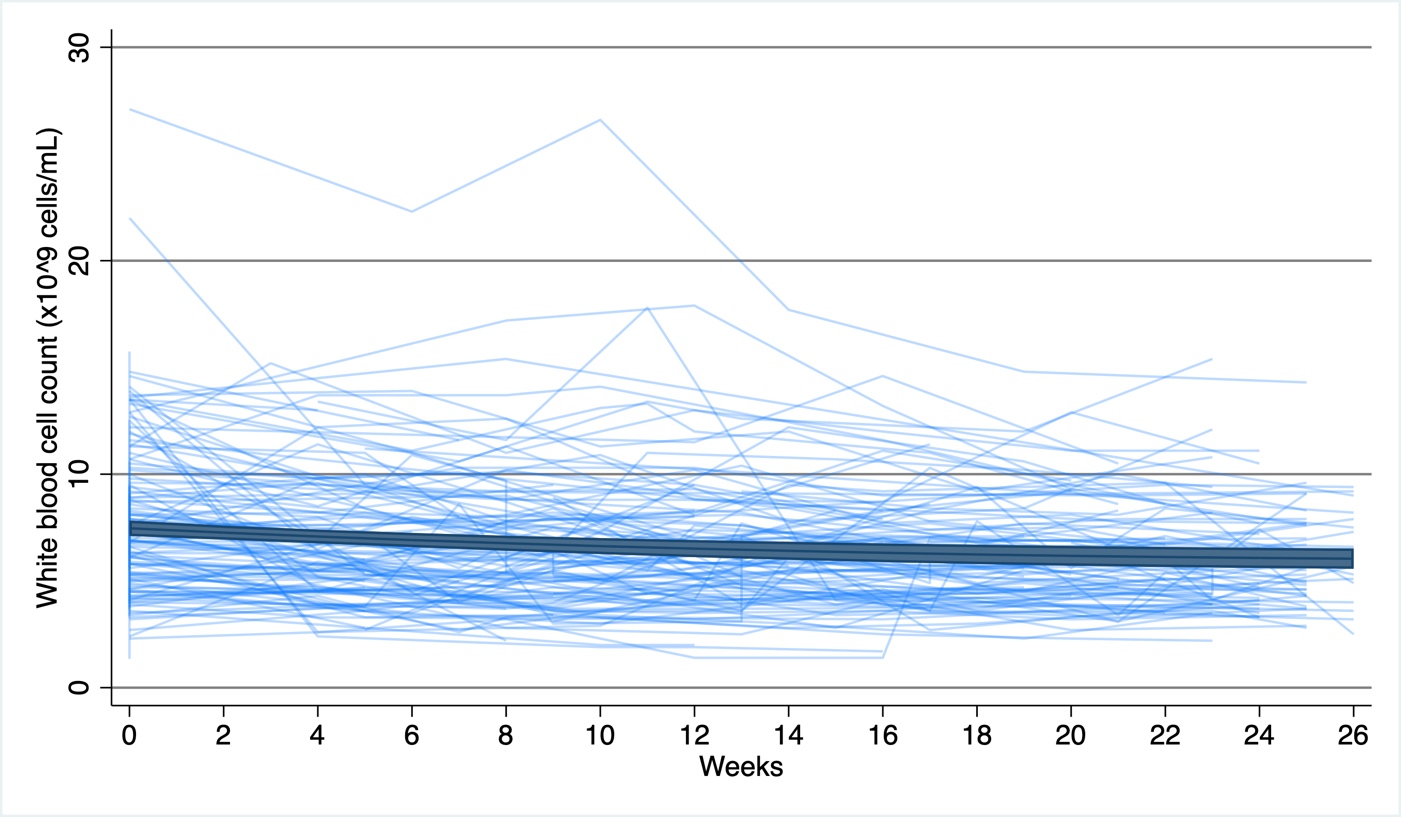
**

**S4 (D). Lactate**

**
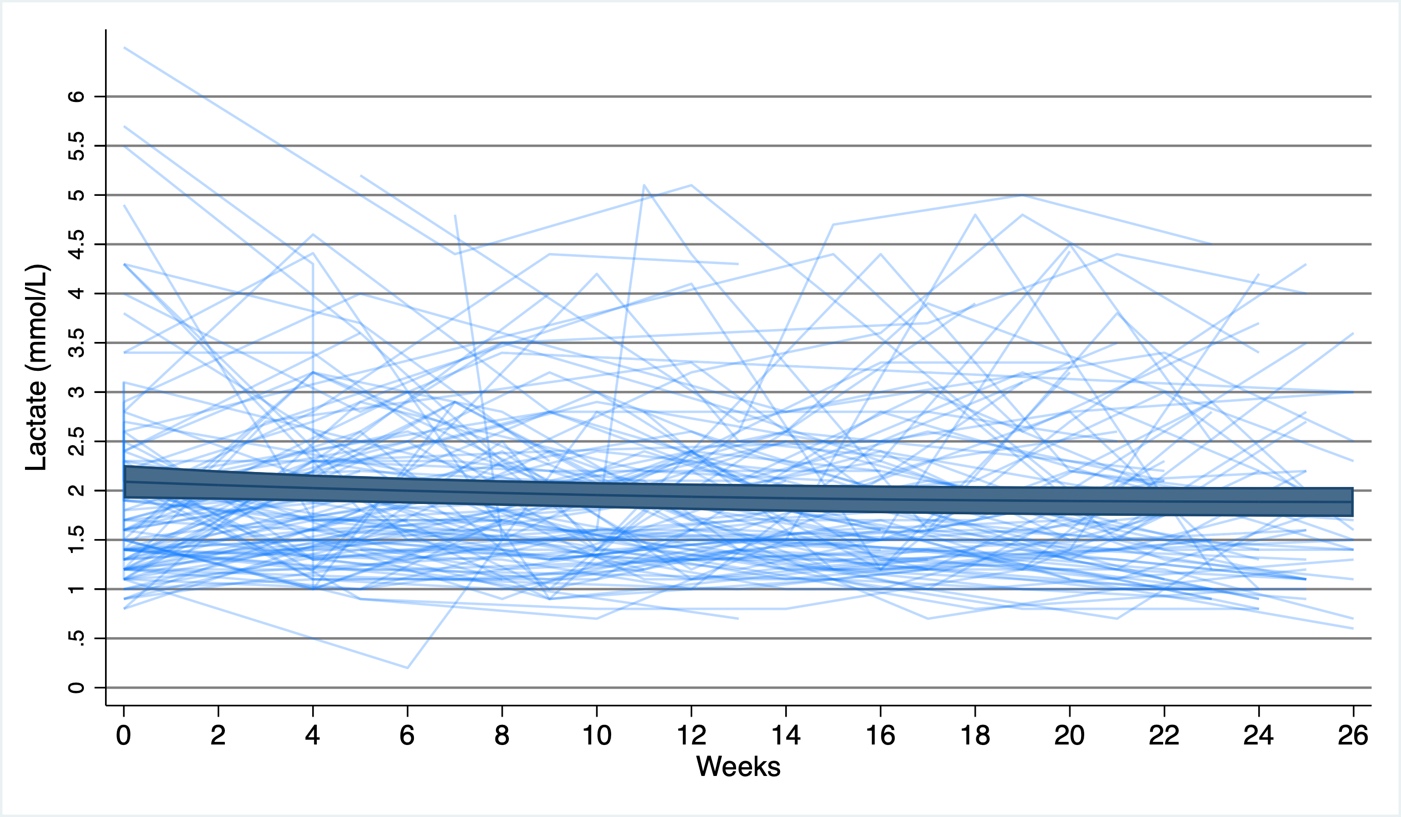
**

**Table S3. Conditional logistic regression model outputs for thrombocytopenia, defined as reduction in platelet count by ≥ 250 x10^9^/L.**

**Table S4. Conditional logistic regression model outputs for hyperlactatemia, defined as increase in lactate concentration by ≥ 1.5 mmol/L.**

**Table S5. Conditional logistic regression model outputs for peripheral neuropathy, defined as any new Grade increase on Brief Peripheral Neuropathy Score.**

**Methods for mitochondrial DNA extraction and sequencing**

*mtDNA yield and quality*

mtDNA was extracted from one of the duplicate samples using the method of Ahmad et al.

2007 for the simultaneous extraction of nDNA and mtDNA from human blood. The samples

were first processed on the bench to separate both the nuclear and cytosol fractions and

mtDNA purification from the cytosolic fraction was then automated using the QIAamp DNA

Blood Mini QIAcube kit on the QIAcube instrument. The yield and purity of the samples were assessed using the NanoDrop-8000 spectrophotometer. mtDNA extractions from the blood samples yielded very low concentrations, and as a result, suboptimal A260/A280 and A260/A230 ratios.Due to the very low concentrations of the mtDNA samples, agarose gel electrophoresis wasnot performed on the samples to assess integrity. Instead, a method of RT-qPCR with SYBR Green I was established to evaluate PCR-amplifiable mtDNA content in the samples. The assay utilized amplification of a 69pb target sequence in the ND1 region (mtND1) (Timken et al. 2005). The validation study of the mtND1 qPCR assay demonstrated specific amplification of a synthetic mtND1-standard, a linear range between 6.0 x10^2^ copies/ul and 6x10^7^ copies/ul and a reaction efficiency of 100.3%. mtND1 was quantified in the samples and the number of copies per microlitre ranged from 5.8 x 10^4^ copies/μL to 4.56 x 10^8^ copies/μL, with a majority of the samples (77%) having > 1.0 x 10^7^ copies/μL thus indicating high content of amplifiable mtDNA in the extracted samples.

*TaqMan OpenArray genotyping*

The mitochondrial nucleotide sequence flanking each SNP (four hundred base pairs upstream and downstream of the SNP site) was retrieved using the UCSC Genome Browser at genome.ucsc.edu. The retrieved sequence was formatted to mask for repeats and the assays were then made using the File Builder software (Life Technologies). All of the assays

were designed so that the allele 1 utilized the VIC probe and the allele 2 utilized the FAM

probe. The SNP assays were purchased preloaded and dried down on TaqMan OpenArray genotyping plates. Each plate is a microscope slide-sized stainless-steel plate that has 3072 through-holes arranged in 48 subarrays of 64 through-holes. Each through-hole acts as a reaction chamber for a TaqMan genotyping assay. The genotyping workflow then consists of loading DNA samples combined (in a 384-well plate) with a genotyping master mix onto OpenArray plates using the QuantStudio 12K Flex OpenArray AccuFill System, followed by thermal cycling and detection using the QuantStudio 12K Flex instrument fitted with an OpenArray block (Applied Biosystems, Life Technologies). The customized SNP assays were run with all submitted samples on the OpenArray plates. NTCs were included as controls. Allele calling was done both automatically by the TaqMan Genotyper Software and manually. Tabular data and Allelic Discrimination plots were generated for reporting.

*mtDNA extraction from frozen blood samples*

The entire blood sample (1-1.5ml) supplied in a tube was transferred to a 15 ml centrifuge

tube and an equal amount of low salt buffer (10mM Tris-HCl pH 7.6, 10mM MgCl2, 10mM KCl,

2mM EDTA) containing 2.5% IGEPAL CA-630 was added and incubated for at least 10 min at

room temperature. This was followed by centrifugation at 800 g for 20 min (Heraeus

Multifuge 3S centrifuge). The supernatant was transferred to a new tube and the residual

nuclear pellet was resuspended in 1.5 ml of buffer with IGEPAL and centrifuged again. The

supernatant from the first and second centrifugation was pooled and centrifuged at 15000g

and at 4 C for 30 min to sediment the mitochondrial pellet. The mitochondrial pellet was washed thrice with the low salt buffer. The pellet was then resuspended in 200 μL lysis buffer

(10mM Tris-HCl pH 7.6, 150mM NaCl, 1% SDS, 2mM EDTA and 0.2mg/ml Proteinase K) and

incubated overnight at 56 C and mtDNA was extracted using the QIAamp DNA Blood Mini

QIAcube kit (QIAGEN) on the QIAcube and eluted in 100 μL.

*QC analysis of purified nDNA samples*

The quality and quantity of the extracted nDNA was measured using the NanoDrop-8000

spectrophotometer. Ratio of the absorbance at 230, 260 and 280 were estimated to assess

the quality and the DNA concentration was measured based on A260 values.

*RT-qPCR for detection of mtDNA*

RT-qPCR assay was performed on ABI QuantStudio 12K Flex Real Time PCR System (Life Technologies, USA). The 10 μL PCR amplification reaction mixtures contained 5 μL of 2  PowerUp SYBR  Green Master Mix (Life Technologies, USA), 0.8 μL of each primer (10 μM), 2.4 μL of nuclease-free water and 1.0 μL of mtDNA. Real-time PCR conditions were: UDG activation at 50 C for 2 min, initial denaturation at 95 C for 2 min, followed by 40 cycles of denaturation at 95 C for 15s, annealing at 52 C for 15s and extension at 72 C for 1 min. All samples were run in duplicate. In each run, an NTC and a standard curve were included. The post-amplification melting curve analysis was performed to confirm whether the nonspecific amplification was generated. The R2 for the standard curve was ≥0.99 and the slope was -3.459.

*TaqMan OpenArray genotyping*

To increase sensitivity for the samples at low concentration, a preamplification protocol was

applied as recommended by the manufacturer. A sample of mtDNA (1.25μL) was mixed with

1.25 μL of a OpenArray PreAmp Pool (specific for the project and ordered at the same time

as the genotyping plates) and 2.5 μL of TaqMan preamplification master mix (Applied Biosystems). The preamplification reaction was performed on an ABI9700 (Applied Biosystems) at the following cycling conditions: single cycle at 95 C for 10 minutes; 14 cycles at 95C for 15 seconds, 60 C for 4 minutes; single cycle at 99 C for 10 minutes in a total volume of 5 μL. Preamplification products were diluted 1:20 in 1 TE buffer. Each diluted preamplified product (2μL) was mixed with 2.0μL of TaqMan OpenArray Genotyping Master Mix in a 384-well plate and mixed. Samples were subsequently loaded onto the OpenArray plate using the QuantStudio 12 K Flex OpenArray AccuFill System. The loaded plates were then sealed and filled with an immersion fluid. PCR amplification was performed on the QuantStudio 12K Flex Real-Time qPCR instrument according to the manufacturer’s recommendations. The genotype profiles for the selected SNPs in each OpenArray plate were determined with the TaqMan Genotyper software version 1.3. The quality value of the data point’s genotype was determined by a threshold above 0.95 and real-time allelic discrimination plots were generated for each assay. Homozygous alleles amplify on the x or y axis, with heterozygous alleles amplifying along the x=y slope.
